# The microRNA Expression Profile in Donation after Cardiac Death (DCD) Livers and Its Ability to Identify Primary Non Function

**DOI:** 10.1371/journal.pone.0127073

**Published:** 2015-05-15

**Authors:** Shirin Elizabeth Khorsandi, Alberto Quaglia, Siamak Salehi, Wayel Jassem, Hector Vilca-Melendez, Andreas Prachalias, Parthi Srinivasan, Nigel Heaton

**Affiliations:** Institute of Liver Studies, King's College Hospital, London, United Kingdom; National Cancer Institute, UNITED STATES

## Abstract

Donation after cardiac death (DCD) livers are marginal organs for transplant and their use is associated with a higher risk of primary non function (PNF) or early graft dysfunction (EGD). The aim was to determine if microRNA (miRNA) was able to discriminate between DCD livers of varying clinical outcome. DCD groups were categorized as PNF retransplanted within a week (n=7), good functional outcome (n=7) peak aspartate transaminase (AST) ≤ 1000 IU/L and EGD (n=9) peak AST ≥ 2500 IU/L. miRNA was extracted from archival formalin fixed post-perfusion tru-cut liver biopsies. High throughput expression analysis was performed using miRNA arrays. Bioinformatics for expression data analysis was performed and validated with real time quantitative PCR (RT-qPCR). The function of miRNA of interest was investigated using computational biology prediction algorithms. From the array analysis 16 miRNAs were identified as significantly different (p<0.05). On RT-qPCR miR-155 and miR-940 had the highest expression across all three DCD clinical groups. Only one miRNA, miR-22, was validated with marginal significance, to have differential expression between the three groups (p=0.049). From computational biology miR-22 was predicted to affect signalling pathways that impact protein turnover, metabolism and apoptosis/cell cycle. In conclusion, microRNA expression patterns have a low diagnostic potential clinically in discriminating DCD liver quality and outcome.

## Introduction

In the climate of organ shortage the donor pool is being expanded by the use of extended criteria/marginal organs as typified by the donation after cardiac death (DCD) liver [1]. The decision making behind the utilization of DCD livers and selecting the appropriate recipient, to achieve optimal outcome is complex. Underpinning all judgements is the assessment of the risk of primary non function (PNF) or early graft dysfunction (EGD). Presently, there is no reliable and objective way to predict DCD EGF or PNF. PNF can be defined as irreversible graft dysfunction requiring retransplant (reLT) within the first 10 days. This manifests with hepatic necrosis, high aspartate transaminase (AST), no bile production, coagulopathy, hypoglycaemia, high lactate and escalating inotropic requirements [2,3]. Whereas with EGD the liver has the potential to recover, but this group is vulnerable to septic complications and prolonged inpatient stay. The reported incidence of PNF is 0–12% and EGD 20–30% [2,3].

The aetiology of graft dysfunction is multifactorial with donor, recipient, preservation and operative factors contributing. The risk of graft dysfunction is highest with DCD livers and typically is associated with a significant ischemia reperfusion injury (I/R). MicroRNAs (miRNAs) are short (21–23 nucleotides) single strands of non-coding RNA that have organ specific and developmental expression with widespread influence on key cellular functions. miRNA is an important control of messenger RNA (mRNA) expression by binding to regulatory sites which are mostly located in the 3′-untranslated region (3’UTR) of mRNA. miRNA control of mRNA is produced either by translational blockade or by affecting mRNA stability leading to its degradation [4]. The aim of this study was to determine if specific miRNA species were associated with DCD livers of varying quality that could be used to identify the risk of PNF.

## Materials and Methods

### Patients and DCD Liver Tissue Samples

From a prospectively maintained database DCD recipients were sequentially identified and divided into 3 groups that were clinically defined, based upon peak serum aspartate transaminase (AST) in the first 5 days after transplant and the need for reLT. The three DCD groups were ‘PNF’ requiring reLT within a week (n = 7), ‘Good’ functional outcome group AST ≤ 1000IU/L (n = 7) and ‘EGD’ AST ≥ 2500 IU/L (n = 9). In total 23 trucut biopsies were analyzed. All samples had been placed in formalin at the time of transplant after perfusion and processed routinely as a formalin fixed paraffin embedded (FFPE) sample. The samples used were all archival and analyzed anonymously. The study had been approved by the Research Committee, Institute of Liver Studies, King's College Hospital. None of the transplant donors were from a vulnerable population and all donors or next of kin provided written informed consent that was freely given. Donor demographics and recipient clinical data are summarized in Table 1.

**Table 1:** Recipient and donor clinical information in the donation after cardiac death groups.

Donor Variables	Good n = 7	EGD n = 9	PNF n = 7	p value
**Donor Liver Weight (g)**	1479 ± 246	1781 ± 346	1770 ± 372	0.2
**WIT (minutes)**	14.7 ± 2.5	16.1 ± 3.9	17.2 ± 4.5	0.4
**Liver Steatosis (%)**	3 ± 5	12 ± 16	7 ± 8	0.3
**Length of stay ICU (days)**	1.6 ± 0.8	3 ± 1.8	4.75 ± 2.6	0.02
**Age (years)**	36 ± 11	43 ± 13	44.4 ± 13.5	0.4
**Sodium (mmol/L)**	148 ± 5	147 ± 9	147 ± 7	0.8
**CIT (minutes)**	400 ± 141	510 ± 215	481 ± 147	0.5
**DRI**	2.3 ± 0.4	2.37 ± 0.6	1.81 ± 0.36	0.2
**Recipient Variables**				
**Recipient Age (years)**	55 ± 3	55 ± 19	49 ± 13	0.6
**Graft Type (whole/reduced)**	7/0	8/1	5/2	-
**MELD**	15 ± 5	9 ± 2	10 ± 12	0.6
**Peak AST (IU/L)**	371 ± 98	9204 ± 5720	11644 ± 4641	0.0001
**Bilirubin day 5 (umol/L)**	79 ± 75	58 ± 26	251 ± 200	0.01
**INR (day 5)**	1 ± 0.09	1.2 ± 0.24	2.5 ± 1.4	0.004
**reLT**	-	-	5	-
**Died after reLT**	-	-	2	-

Summary of recipient and donor clinical information in the three donation after cardiac death (DCD) liver groups of good, early graft dysfunction (EGD) and primary non function (PNF). Abbreviations g (grams), WIT (warm ischemic time), ICU (intensive care unit), CIT (cold ischemic time), DRI (donor risk index), MELD (model for end stage liver disease), AST (serum aspartate transaminase IU/L), INR (international normalised ratio), reLT (redo liver transplant). Where appropriate data expressed as mean and standard deviation.

### RNA extraction

The RNA fraction was isolated from the FFPE biopsies using the High Pure FFPE RNA micro kit (Roche Diagnostics Ltd, Hertfordshire, UK). Six curls at 10μm thickness were taken from the FFPE blocks to which 800μl Xylene was added. Following deparaffinization RNA was isolated according to manufacturer’s recommendations with an overnight incubation at 55°C to increase yields. The Nanodrop 1000 spectrophotometer (Nanodrop Technologies Inc., USA) was used for quantification and the sample stored at -80°C for later analysis.

### High throughput expression analysis: miRNA Arrays

#### Concentrating, labelling and hybridization of samples

Affymetrix GeneChip miRNA 2.0 Arrays (Santa Clara, CA, USA) were used and the standard protocols followed. The total number of mature miRNA probes per array is 15,644 from 131 organisms of which 847 are human (miRBase v15). Probe sets for human snoRNAs were derived from snoRNABAse and Ensembl Archive. In brief, total RNA (tRNA) was enriched using microcon columns (YM-100, Millipore, Watford, UK). 500ng of tRNA was diluted to 100μl with 10mM Tris pH8 and heated at 80°C for 3 minutes, then cooled on ice for 3 minutes. The microcon columns were then calibrated with 50ul of 10mM Tris pH8 and centrifuged (1500 rpm, 3 minutes). Diluted tRNA was then added to the column and centrifuged (13000 g, 7 minutes). RNA was labelled using the FlashTag Biotin RNA Labelling Kit (Genisphere, Hatfield, PA) following manufacturer protocol. GeneChip miRNA 2.0 Arrays (Affymetrix) were then processed using the GeneChip Hybridization, Wash, and Stain Kit with fluidics script FS450_0003. Scanning was performed using Affymetrix Command Console Software. Microarray data generated is MIAME (minimum information about a microarray experiment) compliant and the raw data has been deposited in the gene expression omnibus (accession number: GSE67689).

#### Analysis of scanned miRNA arrays

The array images (CEL files) were processed using Affymetrix miRNA QC Tool, using its Genisphere recommended settings. The workflow includes background correction summarization of multiple probes into an overall probe set intensity with quantile normalization. This procedure generated log2 intensities for the 15,644 probe sets on the chips for each sample. For data analysis and mining Qlucore Omics Explorer (QOE) version 2.1 was used. To determine if there was any intrinsic clustering or outliers in the data unsupervised principal component analysis (PCA) was performed initially. The statistical significance level was set at p = 0.05 with a false discovery rate q value of 0.95. Generation of a heatmap with QOE v2.1 enabled further visualization of the data. For multigroup comparison F test based on ANOVA was used. miRNAs that were significantly (p < 0.05) differentially expressed between the DCD groups was then selected for further study. As the Affymetrix GeneChip miRNA 2.0 Arrays covers 131 different organisms, if there was no miRNA sequence homology with the human counterpart it was excluded from further consideration. Table 2 summarizes the miRNAs that were selected for study.

**Table 2:** Summary of microRNA with differential expression between the donation after cardiac death groups.

miRNA	Fold Change	Accession number	Biological Role
	(p<0.05)	Chromosome	
	PNF vs Good + EGD	Sequence	
hsa-miR-107	-1.8 (p = 0.02)	MIMAT0000104	Insulin Sensitivity, inhibits
		10: 91,350,504–91,354,584	HIF1, cell cycle arrest, cancer
		50—agcagcauuguacagggcuauca—72	stem cell phenotype
hsa-miR-378	-1.6 (p = 0.04)	MIMAT0000731	Cell survival,
		5: 149,110,388–149,114,453	apoptosis/proliferation
		5—cuccugacuccagguccugugu—26	balance
hsa-miR-23b	-2.6 (p = 0.02)	MIMAT0004587	Termination of Liver
		9: 97,845,490–97,849,586	Regeneration
		20—uggguuccuggcaugcugauuu—41	
hsa-miR-122_st	-6 (p = 0.02)	MIMAT0000421	Mitochondrial Function, cell
		18: 56,116,306–56,120,390	cycle arrest, fatty acid
		15—uggagugugacaaugguguuug—36	metabolism, specific to liver (72% of total miRNA in liver)
hsa-miR-103	-2.2 (p = 0.02)	MIMAT0000101	Related to miR107 roles,
		5: 167,985,901–167,989,978	involved in hypoxia and
		48—agcagcauuguacagggcuauga—70	insulin sensitivity
hsa-mir-125b	-1.8 (p = 0.03)	MIMAT0004592	Cell cycle arrest, cell
		11: 121970465–121970552	proliferation, anti-apoptotic
		15—ucccugagacccuaacuuguga- 36	
hsa-miR-24	-2 (p = 0.04)	MIMAT0000080	Proliferation, apoptosis
		19: 13947101–13947173	
		50—uggcucaguucagcaggaacag- 71	
hsa-miR-let-7a-5p	-1.6 (p = 0.03)	MIMAT0000062	Developmental timing,
		22:46506629–46510702	differentiation of stem cell
		4—ugagguaguagguuguauaguu—25	lineage, highly conserved across species, abundant expression liver
hsa-miR-191	-1.5 (p = 0.02)	MI0000465	Senescence (growth arrest
		3: 49058051–49058142	during which cells remain
		16—caacggaaucccaaaagcagcug- 38	metabolically active), epithelial mesenchymal
			transformation
hsa-miR-194	-1.6 (p = 0.4)	MIMAT0000460	Inhibits epithelial
		1: 220291499–220291583	mesenchymal
		15—uguaacagcaacuccaugugga- 36	transformation, hepatocyte abundant
hsa-miR-296-5p	-1.6 (p = 0.02)	MIMAT000690	Angiogenesis and modulate
		20:57,395,281–57,391,368	embryonic stem cell
		48—gaggguuggguggaggcucucc- 69	differentiation
hsa-miR-455-3p	-1.6 (p = 0.03)	MIMAT0004784	
		9: 116,969,714–116,973,809	
		54—gcaguccaugggcauauacac—74	
hsa-miR-940	-2 (p = 0.01)	MIMAT0004983	
		16:2,323,748–2,319,841	
		60—aaggcagggcccccgcucccc- 80	
hsa-miR-let-7d-5p	-1.5 (p = 0.03)	MIMAT0000065	See let-7a entry
		9:96,943,116–96,939,202	
		8—agagguaguagguugcauaguu—29	
hsa-miR-22	-1.6 (p = 0.047)	MIMAT0000077	Apoptosis, Hypoxia Signalling
		17: 1617197–1617281	(see main discussion)
		53—aagcugccaguugaagaacugu- 74	
hsa-miR-155	-2 (p = 0.01)	MIMAT0000646	Immunity (adaptive/innate),
		21: 26946292–26946356	apoptosis, HIF-1
		4—uuaaugcuaaucgugauaggggu -26	

Summary of microRNA (miRNA) species found to be have significant differential expression between the three donation after cardiac death (DCD) liver groups based on initial analysis of the array data. Expressed as fold change in miRNA in primary non function (PNF) group compared to the good and early graft dysfunction (EGD) group as calculated with Qlucore Omics Explorer v2.1 (p<0.05). Additional miRNA data derived from miRBase and Ensembl for the miRNA mature sequence, annotations and chromosomal locations. Potential biological role of miRNA relevant to the DCD liver reported in the literature (in vitro and in vivo) is also summarized.

#### Validation of miRNA expression: Real time quantitative PCR (RT-qPCR)

RT-qPCR was performed to validate the miRNA that the array analysis had identified as having significant differential expression between the DCD groups. The miRCURY LNA Universal real-time microRNA PCR system was used according to the manufacturer’s protocol (EXIQON Inc. USA). In brief, first strand cDNA was synthesized from 20ng of template RNA to create a miRNA cDNA library. Real time PCR amplification was then performed using SYBR Green master mix (EXIQON Inc. USA), forward and reverse primers for each miRNA species (EXIQON Inc. USA) and ROX reference dye (Invitrogen). The PCR reactions were performed on an Applied Biosystems 7900HT Fast Real Time PCR machine (Life Technologies Corporation, CA, USA). In each assay, 1 μ L of cDNA was used for amplification PCR cycle of fast condition enzyme activation 20 seconds at 95°C, melt 3 seconds at 95°C, anneal/extend 30 seconds at 60°C, with an added dissociation stage, cycle 40 x. The PCR reactions were carried out in a final volume of 22ul and performed in triplicate.

The relative quantitation data was exported for analysis. Initial data analysis was performed with manufacturer software provided with Applied Biosystems 7900HT Fast Real Time PCR machine (Life Technologies Corporation, CA, USA) to obtain threshold cycle (Ct) value that was determined using default threshold settings. To correct for potential RNA input or RT efficiency biases Ct values were normalised using the average Ct of the endogenous control. Small nucleolar RNAs (snoRNAs), hsa-SNORD49A and hsa-SNORA66, were used as endogenous reference RNA. For miRNA relative expression analysis 2ΔΔCT was used and calculated as follows ΔCT (miRNA Ct—averaged endogenous control Ct) and fold-change to reference sample of normal liver. ‘Normal liver’ was a sample from a non steatotic donation after brain stem (DBD) liver. Due to the range and magnitude of relative miRNA expression, data was log transformed and presented as mean +/- SE. Intergroup comparisons were performed with ANOVA and appropriate post hoc test to compensate for multiple comparisons.

### Computational Biology

A number of publically available databases were explored to characterize the miRNA and to explore putative proteins regulated by a given miRNA. For naming, annotation and published miRNA sequences mirBase database v12 was used. For target analysis and gene ontology clustering GOmir was used [5]. GOmir intergrates miRNA target prediction and functional analyses by combining the predicted target genes of TargetScan, miRanda, RNAhybrid and PicTar using computerised prediction algorithms supplemented by the experimentally supported targets from TarBase. To look at pathways affected by a given miRNA the prediction software of DIANA-TarBase 6.0 (microT-4) for KEGG (Kyoto Encyclopedia of Genes and Genomes) pathways enrichment was used. KEGG is a computerised representation of a biological system and a KEGG pathway is a collection of manually drawn pathway maps.

## Results

### Donor and Recipient Demographics

Table 1 summarizes the characteristics of the donor, recipient and transplanted DCD liver. Overall the DCD livers had short warm ischemic times (<30 minutes), were non or mildly steatotic (<30%) and the cold ischemic time was normally < 8 hours. The only factor from the donor variables that was significantly different between the DCD groups was the donor length of stay in the intensive care unit. Calculation of the donor risk index did not demonstrate any significant differences between the DCD groups.

### Differentially expressed miRNA from array analysis

**Fig 1:**
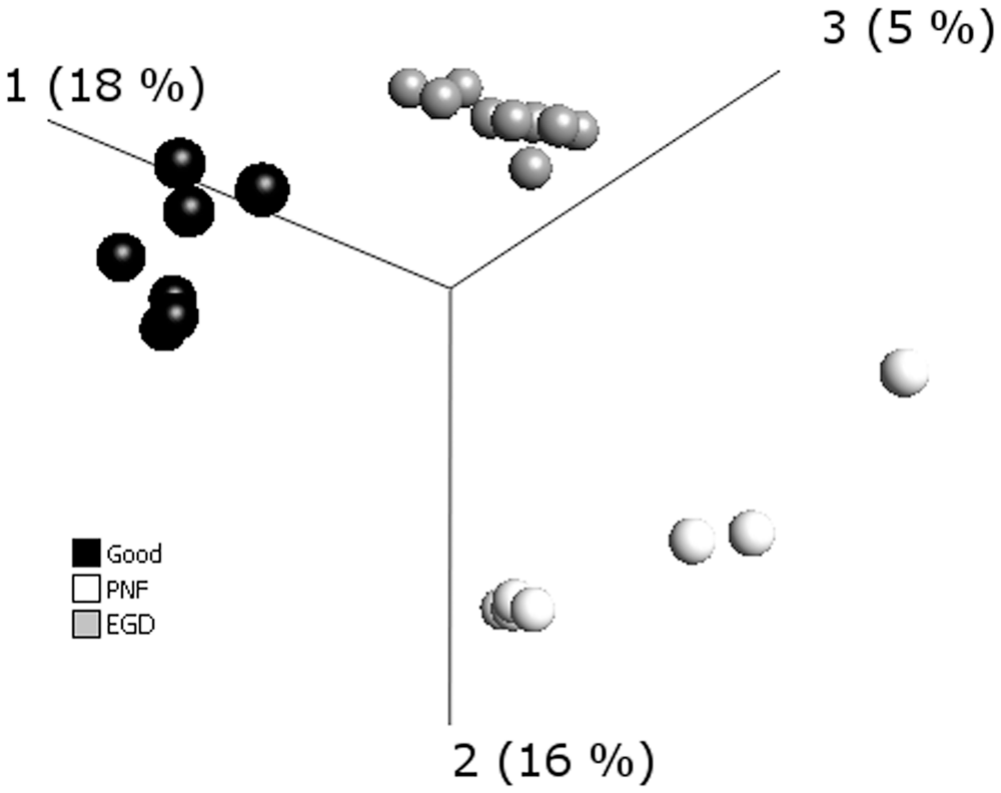
Principal Component Analysis (unsupervised hierarchial cluster analysis) of microRNA (miRNA) array data. miRNA was extracted from archival formalin fixed post-perfusion tru-cut donor liver biopsies taken from donation after cardiac death (DCD) livers of varying clinical outcome. DCD groups were categorized as primary non function (PNF) retransplanted within a week (n = 7), good functional outcome (n = 7) peak aspartate transaminase (AST) ≤ 1000 IU/L and early graft dysfunction (EGD) (n = 9) peak AST ≥ 2500 IU/L. The principal component analysis shows clustering of samples according to DCD liver group of PNF, EGD and good (p = 0.05, q = 0.95).

**Fig 2:**
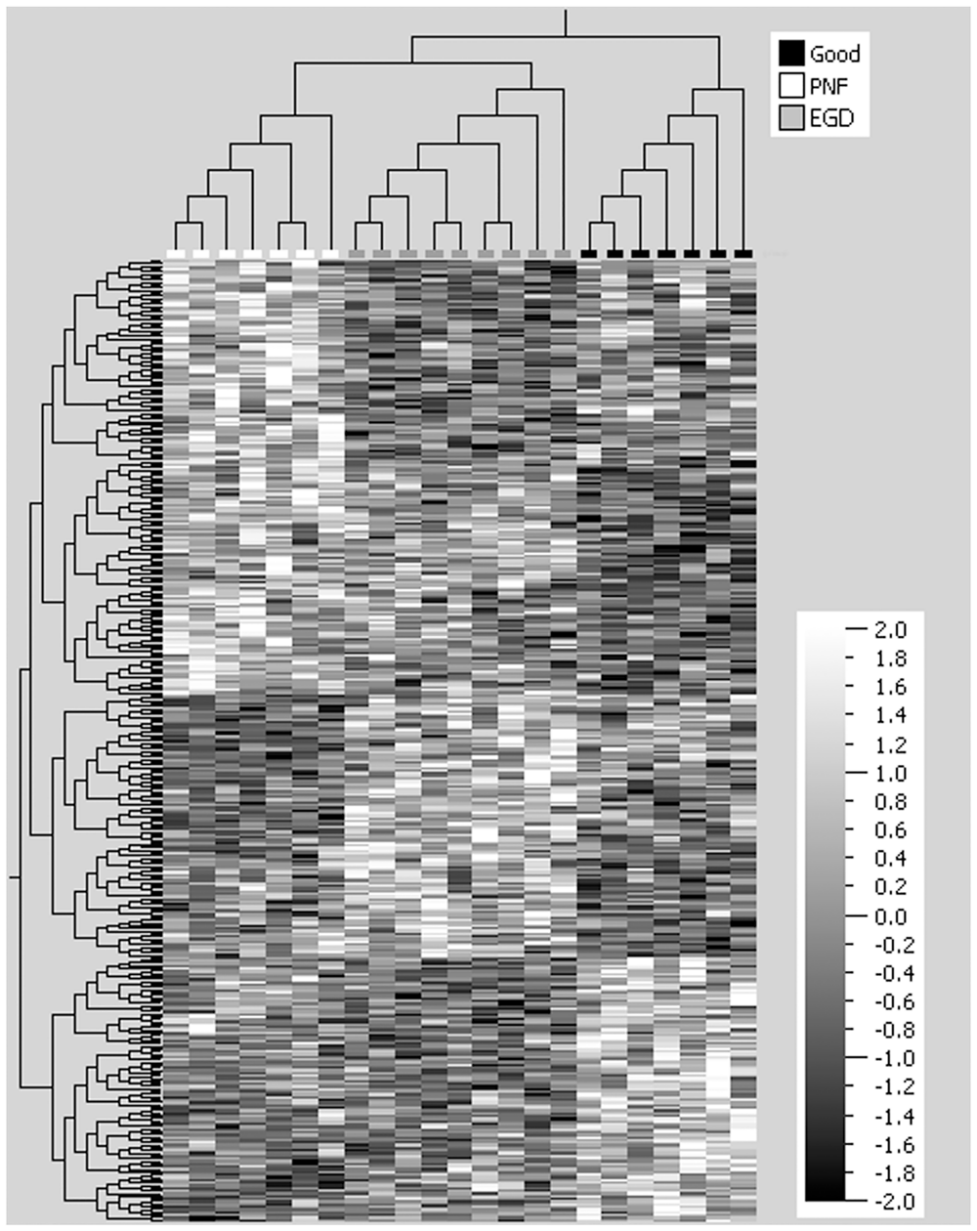
Heatmap of microRNA from the donation after cardiac death liver groups. microRNA (miRNA) was extracted from archival formalin fixed post-perfusion tru-cut donor liver biopsies taken from donation after cardiac death (DCD) livers of varying clinical outcome. DCD groups were categorized as primary non function (PNF) retransplanted within a week (n = 7), good functional outcome (n = 7) peak aspartate transaminase (AST) ≤ 1000 IU/L and early graft dysfunction (EGD) (n = 9) peak AST ≥ 2500 IU/L. The heatmap demonstrates miRNA differential expression between the three DCD liver groups of primary PNF, EGD and good (p<0.05). Columns of the heatmap represent the different individual DCD liver biopsies and the horizontal cladogram at the top of the heatmap demonstrates clustering of samples according to DCD group of PNF, EGD and good. Key in top corner illustrates color labelling of DCD groups in the horizontal cladogram. The rows of the heatmap represent different miRNAs and the vertical cladogram shows clustering of miRNA species within each DCD group. The vertical graded scale shows that white within the heatmap represents increased expression and black decreased expression of a given miRNA. As no microRNA species was strongly associated with a given DCD clinical group, there is no clear heatmap pattern to be seen.

After normalization and data filtering, the miRNAs from the DCD livers analysed was resolved into 3 principal components as represented by the 3 dimensional scatter plot of the PCA (Fig 1). The PCA, an unsupervised hierarchial cluster analysis (HCA), illustrates clustering of samples according to the DCD groups of PNF, EGD and Good (p = 0.05, q = 0.95). Subsequent supervised HCA, generating a heatmap, confirmed that the miRNA expression profiles were similar within each DCD group (Fig 2). The graphical tree (cladogram) above the heat map (Fig 2) also illustrates the clustering of samples according DCD group. Candidate miRNAs for RT-qPCR validation were then chosen from the QOE v2.1 generated list of miRNAs identified to have significant (p<0.05) differential expression between the DCD groups. Table 2 summarizes miRNAs that were selected for further study.

### RT-qPCR validation and quantification of candidate miRNA

**Fig 3:**
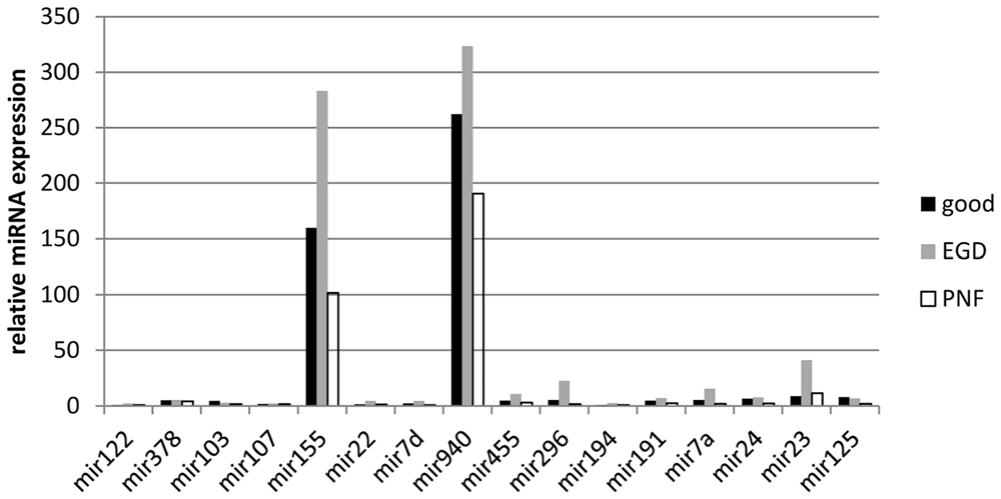
Real time quantitative PCR profile in donation after cardiac death liver. From the microRNA (miRNA) array analysis 16 miRNAs were identified as having significant differential expression (p<0.05) and selected for validation with real time quantitative PCR (RT-qPCR). Threshold cycle (Ct) values were normalised using the average Ct of small nucleolar RNAs (snoRNAs) and to determine miRNA relative expression 2ΔΔCT was used and calculated as follows ΔCT (miRNA Ct—averaged endogenous control Ct) and fold-change to reference sample of normal liver (a non steatotic donation after brain stem death liver). Across the three donation after cardiac death (DCD) liver groups of primary non function (PNF) retransplanted within a week (n = 7), good functional outcome (n = 7) peak aspartate transaminase (AST) ≤ 1000 IU/L and early graft dysfunction (EGD) (n = 9) peak AST ≥ 2500 IU/L, miR-155 and miR-940 had the highest relative expression.

**Fig 4:**
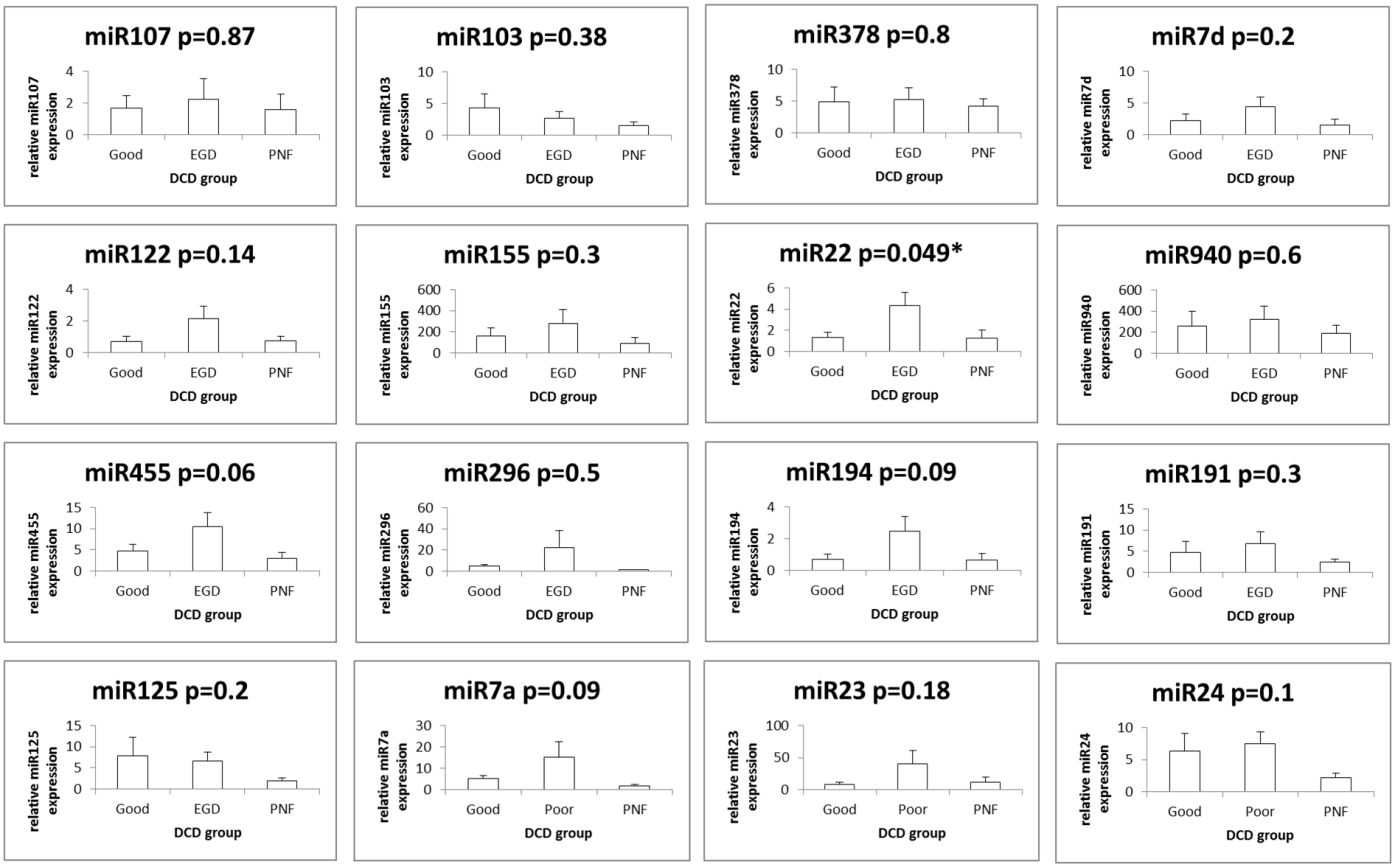
Real time quantitative PCR of individual microRNA. From the microRNA (miRNA) array analysis 16 miRNAs were identified as having significant differential expression (p<0.05) and selected for further validation with real time quantitative PCR (RT-qPCR). Threshold cycle (Ct) values were normalised using the average Ct of small nucleolar RNAs (snoRNAs) and to determine miRNA relative expression 2ΔΔCT was used and calculated as follows ΔCT (miRNA Ct—averaged endogenous control Ct) and fold-change to reference sample of normal liver (a non steatotic donation after brain stem death liver). Due to the range and magnitude of relative miRNA expression, data was log transformed and presented as mean +/- SE. The relative expression for a given miRNA in the three donation after cardiac death (DCD) liver groups of primary non function (PNF) retransplanted within a week (n = 7), good functional outcome (n = 7) peak aspartate transaminase (AST) ≤ 1000 IU/L and early graft dysfunction (EGD) (n = 9) peak AST ≥ 2500 IU/L is represented graphically. The observed differences in the relative expression of a given miRNA species on RT-qPCR between the three DCD liver groups was only found to be significant for miR-22 (p = 0.049).

miRNAs with overall high relative expression in all three clinical DCD liver groups were miR-940 and miR-155. All other miRNAs (miR-107, miR-378, miR-236, miR-122_st, miR-103, miR-125b, miR-24, let-7a, miR-191, miR194, miR-296-5p, miR-455-3p, let-7d and miR-22) had a low level of relative expression. Fig 3 illustrates the relative expression of these miRNAs. Fig 4a and 4b illustrates the RT-qPCR miRNA expression profiles between the three DCD groups of each miRNA analyzed. The difference in relative expression of miRNA on RT-qPCR between the three DCD groups was only found to be significant for miR-22 (p = 0.049, ANOVA post-hoc tests: Good vs EGD: Diff = 2.9690, 95%CI = -0.5268 to 6.4648, p = 0.1057, Good vs Poor: Diff = -0.1500, 95%CI = -3.5540 to 3.2540, p = 0.9932, EGD vs Poor: Diff = -3.1190, 95%CI = -6.4012 to 0.1632, p = 0.0645).

### Computational biology prediction for hsa-miR-22

On GOmir analysis 38 overlapping gene targets were identified for miR-22, of which 26 were of potential biological relevance in the transplanted liver and are listed in S1 Table, being selected on the basis of their cellular function. The majority of the gene targets encoding for proteins that impact signalling cascades, ATP turnover and code replication. KEGG Pathways enrichment of miR-22 target genes using prediction software microT-3 set at a loose score threshold identified 221 target genes that are involved in 50 pathways. The KEGG pathways of potential biological relevance in the present model of discriminating DCD liver quality and outcome are listed in S2 Table. The computationally predicted miR-22 regulated predicted genes have a preponderance in signalling pathways that impact protein turnover, metabolism and apoptosis/cell cycle.

## Discussion

Despite all the advances in liver transplant assessing the donor liver for its ‘transplantability’, remains highly subjective and experience driven. Being able to objectively assess the liver in addition to gaining insight into its molecular pathophysiology has the potential to optimize the use of marginal livers and improve clinical outcome in the recipient [6]. The present understanding of PNF is that it is a ‘reperfusion’ injury resulting in irreversible graft failure with no associated technical or immunological problems [3]. The understanding of what occurs with graft recovery after ischemia reperfusion remains poorly understood. Elements that are recognized to contribute include the liver’s metabolic capacity, degree of cellular injury and regenerative potential.

miRNAs were discovered in 1993, they are highly conserved, have ubiquitous expression and are acknowledged to be important regulators of gene expression. Each miRNA is predicted to regulate several kinds of gene and modulate/fine tune almost all biological processes [4,7,8]. Normal liver has been shown to be rich in miR-let-7a, miR-122 and miR-21, with miR-130a, miR-130b, miR-140*, miR-320, miR-671 localizing to the mitochondria [9,10]. In liver regeneration up regulation of miR-21 [11–14] and down regulation of miR-150, miR-663 and miR-503 [15] have been identified as being of importance. While in mouse models of liver ischemia/reperfusion increased expression of miR-326 and miR-223 have been found, and miR-23a, miR-346 and miR-370 levels correlate with serum AST value [16].

The literature on miRNAs and assessment of donor liver quality is limited [6]. What has been shown is that there is a higher ratio of selected hepatocyte miRNAs (miR-122, miR-148a) to cholangiocyte miRNAs (miR-30e, miR-222, miR-296) in liver perfusate from recipients with biliary complications [17]. While low levels of miR-122 and miR-148a in post perfusion DCD liver biopsies are observed as their serum levels increase, correlating with warm ischemic time (WIT) but having no association with cold ischemic time (CIT) [18].

From the DCD miRNA expression data in this present work miR-940 and miR-155 were consistently found to have the highest expression, across all DCD groups of good, EGD and PNF, instead of the typical liver profile of miR-122 and let-7 family member expression dominating [9,10]. miR-155 has been demonstrated to have a regulatory role in apoptosis, the innate immune system and hypoxia inducible factor 1 expression (HIF1) [19]. HIF1 is a pivotal transcription factor that responds to changes in cellular oxygen levels to orchestrate changes in gene expression [20]. In contrast, the biological role of miR-940 has not been experimentally validated, but from computational biology it has a predicted role influencing metabolic pathways and oxidative phosphorylation.

Overall, the difference in the miRNA expression between the DCD groups of PNF, EGD and good was subtle, with only one miRNA (miR-22) appearing to distinguish between groups. miRNA is regarded as a fine tuner of mRNA expression, so a dramatic change in miRNA expression may not necessarily be needed to produce substantial changes in biology, as a small change in miRNA expression can facilitate rapid translational repression and/or degradation of hundreds of target genes [21]. Emerging data is also demonstrating that the rate of recruitment of miRNA to the RNA induced silencing complex (RISC) is of importance in determining the degree of target suppression and that a change in miRNA expression is not necessarily required to alter mRNA expression [22].

Another aspect is most miRNA studies are on differential expression at a fixed time point, with little attention being given to miRNA temporal kinetics [7,23]. This maybe of relevance in distinguishing DCD liver quality as different biological scenarios can lead to alternative response programs or miRNome’s that lead to a cyclical, sustained or peaked response [7]. In the context of a DCD liver, a sequence of overlapping cellular response programmes are being triggered by warm ischemia in the donor, cold ischemia during storage/transportation then reperfusion in the recipient. This may explain the lack of a distinctive and clear miRNome response being identified for PNF in the data we have presented [24].

The finding that miR-22 expression may be of use in distinguishing a DCD liver with PNF from that of EGD, which has the potential to recover, could be biologically plausible. Emerging data from animal models of cellular hibernation show that there is an established sequence of events. When initiated cellular hibernation results in what is described as the ‘survival response’ that alters cell survival, cell cycle, glucose metabolism and protein translation [25]. The primary objective of this ‘survival response’ is to minimise cellular replacement processes in order to facilitate longterm survival and this is epitomized by cellular proliferation arrest without triggering apoptosis. miRNA is ideally placed to give an additional layer of regulation to the rapid and reversible fluctuations in the transcriptome for the cellular survival response. From the computational biology analysis miR-22 is in the position to regulate a number of pathways that could be relevant to the survival response such as cell cycle, metabolism and kinase signalling [25] (see S1 and S2 Tables).

The anti-proliferative effect of miR-22 has only been demonstrated in a number of cancer models [26]. From studies on cancer cells, miR-22 has been demonstrated to affect HIF-1 [27] and Phosphatase and Tensin homolog (PTEN) activity/expression [28,29]. HIF-1 is pivotal for the hypoxic response [20] and is a key player in determining apoptosis/proliferation balance, as is the PTEN/PI3K/Akt pathway. These roles of miR-22 maybe relevant in the DCD liver, as miR-22 could help shift cell priority to survival by blocking apoptosis, but avoiding the initiation of cell proliferation. The apoptosis/proliferation balance could be critical in the immediate period after transplant in order to conserve energy, then after this period of recovery the DCD liver is able to enter a period of regeneration [15]. Therefore, the ability of a DCD liver to switch to ‘survival mode’ may determine its fate of being a liver that is able to recover (EGD) to one that is not (PNF).

In summary, microRNA expression patterns appear to have a low diagnostic potential clinically in discriminating DCD liver quality and outcome. However, more study is required to determine whether the ‘survival response’ and miR-22 are important determinants of outcome in DCD liver transplantation.

## Supporting Information

S1 Table GOmir analysis of microRNA 22. Summary of GOmir analysis of microRNA 22 (miR-22) predicted gene targets of potential biological relevance in determining outcome in donation after cardiac death (DCD) liver transplantation of early graft dysfunction (EGD) or primary non function (PNF). GOmir combines the predicted target genes of TargetScan, miRanda, RNAhybrid and PicTar using computerised prediction algorithms supplemented by the experimentally supported targets from TarBase.
(DOCX)

S2 Table Predicted targets of microRNA 22. Summary of genes and pathways affected by a microRNA 22 using the prediction software of DIANA-TarBase 6.0 (microT-4) for KEGG (Kyoto Encyclopedia of Genes and Genomes) pathways enrichment.
(DOCX)
