# Supplementary material for: The microRNA Expression Profile in Donation after Cardiac Death (DCD) Livers and Its Ability to Identify Primary Non Function
Source: PLoS One. 2015 May 15;10(5):e0127073. doi: 10.1371/journal.pone.0127073 (PMC4433116; doi:10.1371/journal.pone.0127073)
Supplement: S1 Table — Summary of GOmir analysis of microRNA 22 (miR-22) predicted gene targets of potential biological relevance in determining outcome in donation after cardiac death (DCD) liver transplantation of early graft dysfunction (EGD) or primary non function (PNF). GOmir combines the predicted target genes of TargetScan, miRanda, RNAhybrid and PicTar using computerised prediction algorithms supplemented by the experimentally supported targets from TarBase. (DOCX) [file pone.0127073.s001.docx]

| **Abbreviation** | **Protein Name** | **Function** |
| --- | --- | --- |
| **AKT3** | **AKT serine/threonine protein kinase family** | cell signaling in response to insulin and growth factors, involved in cell proliferation, differentiation, apoptosis, tumorigenesis, as well as glycogen synthesis and glucose uptake. |
| **ARFIP2** | **ADP-ribosylation factor interacting protein 2** | GTP binding protein of Ras superfamily involved in vesicular intracellular traffic |
| **BRSK2** | **BR serine/threonine kinase 2** | related to AMP activated protein kinase involved in energy homeostasis |
| **BTG1** | **B-cell translocation gene 1, anti-proliferative gene** | Immediate early gene, interacts with general transcription complex, transcription factors and modulates intracellular signal transduction cascades |
| **CHD9** | **Chromodomain-helicase-DNA-binding protein 9** | ATP dependent chromatin remodeling, interacting partner determines whether repressor/activator |
| **COPS7B** | **COP9 signalosome complex subunit 7b,** | related to 26S ribosome regulatory complex |
| **EPC1** | **Enhancer of polycomb homolog 1** | transcriptional repressor |
| **ERBB3** | **Receptor tyrosine-protein kinase erbB-3** | member of the epidermal growth factor receptor (EGFR) family of receptor tyrosine kinases, heterodimerization leads to cell proliferation |
| **FBXW7** | **F-box/WD repeat-containing protein 7** | F-box proteins constitute one of the four subunits of ubiquitin protein ligase complex called SCFs (SKP1-cullin-F-box), which function in phosphorylation-dependent ubiquitination |
| **IPO7** | **Importin-7** | prevents the activation of Ran's GTPase by RanGAP1 and inhibits nucleotide exchange on RanGTP, and also binds directly to nuclear pore complexes |
| **JARID2** | **Jumonji, AT rich interactive domain 2** | regulator of cell proliferation |
| **MECP2** | **Methyl CpG binding protein 2** | repressing/silencing other genes |
| **NDEL1** | **Nuclear distribution protein nudE-like 1** | binds strongly to [centrosomes](http://en.wikipedia.org/wiki/Centrosome) in [interphase](http://en.wikipedia.org/wiki/Interphase) and localized to [mitotic spindles](http://en.wikipedia.org/wiki/Mitotic_spindle) in early M phase. |
| **PDSS1** | **Decaprenyl-diphosphate synthase subunit 1** | an enzyme that elongates the prenyl side-chain of coenzyme Q, or ubiquinone, one of the key elements in the respiratory chain |
| **PTPN9** | **Tyrosine-protein phosphatase non-receptor type 9** | signaling molecule involved in cell growth, differentiation, mitotic cycle |
| **RBM15** | **Putative RNA-binding protein 15** | suppressor function |
| **RGS2** | **Regulator of G-protein signaling 2** | family member of G protein coupled receptor |
| **SIRT1** | **sirtuin (silent mating type information regulation 2 homolog) 1** | deacetylates proteins that contribute to cellular regulation (reaction to stressors, longevity) |
| **SLC2A1** | **Solute carrier family 2** | facilitated glucose transporter, member 1 or GLUT1 |
| **STK39** | **STE20/SPS1-related proline-alanine-rich protein kinase** | function in the cellular stress response pathway |
| **TLK2** | **Serine/threonine-protein kinase tousled-like 2** | nuclear serine/threonine kinases involved in the regulation of chromatin assembly |
| **TRIB2** | **Tribbles homolog 2** | regulates MAPK (mitogen activated protein kinases) signaling pathways |
| **TYRO3** | **Tyrosine-protein kinase receptor** | interacts with the PI3 kinases (phosphatidylinositide-3-kinase) signaling pathway and GAS6 (growth arrest specific 6) involved in cell proliferation |
| **WDTC1** | **WD and tetratricopeptide repeats 1** | highly conserved, regulate cellular functions, such as cell division, cell fate, transcription, transmembrane signalling |
| **ZFYVE9** | [**Zinc finger FYVE domain-containing protein 9**](http://en.wikipedia.org/wiki/Zinc_finger_FYVE_domain-containing_protein_9) | anchoring protein involved in TGF beta signaling, phosphorylating transcription factors |
