# Supplementary material for: The microRNA Expression Profile in Donation after Cardiac Death (DCD) Livers and Its Ability to Identify Primary Non Function
Source: PLoS One. 2015 May 15;10(5):e0127073. doi: 10.1371/journal.pone.0127073 (PMC4433116; doi:10.1371/journal.pone.0127073)
Supplement: S2 Table — Summary of genes and pathways affected by a microRNA 22 using the prediction software of DIANA-TarBase 6.0 (microT-4) for KEGG (Kyoto Encyclopedia of Genes and Genomes) pathways enrichment. (DOCX) [file pone.0127073.s002.docx]

| **KEGG PW** | **GENE NAME** | **FOUND GENES** | **-ln (p value)** |
| --- | --- | --- | --- |
| MAPK signaling pathway | NTRK2, TGFBR1, FLNA, NR4A1, MAP3K3, DUSP10, MAP3K14 | 7 | 3.27 |
| Ubiquitin mediated proteolysis | UBE4A, CBL, ANAPC5 | 3 | 0.78 |
| p53 signaling pathway | CDK6, SESN1 | 2 | 0.76 |
| Apoptosis | TNFRSF10D, MAP3K14 | 2 | 0.46 |
| Proteasome | PSMB2 | 1 | 0.4 |
| ErbB signaling pathway | CBL | 1 | 0.38 |
| Phosphatidylinositol signaling system | PIP4K2B | 1 | 0.38 |
| PPAR signaling pathway | PPARA | 1 | 0.35 |
| Calcium signaling pathway | ADCY7, ADORA2A | 2 |  |
| Jak-STAT signaling pathway | CBL | 1 | 0.22 |
| Hedgehog signaling pathway | PTCH1 | 1 | 0.18 |
| Wnt signaling pathway | PSEN1 | 1 | 0.17 |
| Purine metabolism | AK2, ADCY7 | 2 | 0.14 |
| Insulin signaling pathway | CBL | 1 | 0.12 |
| Inositol phosphate metabolism | PIP4K2B | 1 | 0.09 |
| Cell cycle | CDK6, ANAPC5 | 2 | 0.08 |
| Oxidative phosphorylation | ATP6V1A | 1 | 0.04 |
| Aminoacyl-tRNA biosynthesis | YARS | 1 | 0.04 |
| Notch signaling pathway | PSEN1 | 1 | 0 |
| Hedgehog signaling pathway | PTCH1 | 1 | 0.18 |
